# Organic Transistors Based on Highly Crystalline Donor–Acceptor π-Conjugated Polymer of Pentathiophene and Diketopyrrolopyrrole

**DOI:** 10.3390/molecules29020457

**Published:** 2024-01-17

**Authors:** Shiwei Ren, Zhuoer Wang, Jinyang Chen, Sichun Wang, Zhengran Yi

**Affiliations:** 1 Zhuhai-Fudan Research Institute of Innovation, Guangdong-Macao In-Depth Cooperation Zone, Hengqin 519031, China; shiwei_ren@fudan.edu.cn; 2 Department of Materials Science, Fudan University, Shanghai 200438, China; 3 Technical Center of Gongbei Customs District, Zhuhai 519001, China; 4 Key Laboratory of Colloid and Interface Chemistry of Chemistry and Chemical Engineering, Shandong University, Jinan 250100, China; wangzhuoer94@163.com; 5 Zhejiang Key Laboratory of Alternative Technologies for Fine Chemicals Process, Shaoxing University, Shaoxing 312000, China

**Keywords:** organic electronics, solution processing, donor–acceptor, π-conjugated polymer material, hole mobility

## Abstract

Oligomers and polymers consisting of multiple thiophenes are widely used in organic electronics such as organic transistors and sensors because of their strong electron-donating ability. In this study, a solution to the problem of the poor solubility of polythiophene systems was developed. A novel π-conjugated polymer material, PDPP-5Th, was synthesized by adding the electron acceptor unit, DPP, to the polythiophene system with a long alkyl side chain, which facilitated the solution processing of the material for the preparation of devices. Meanwhile, the presence of the multicarbonyl groups within the DPP molecule facilitated donor–acceptor interactions in the internal chain, which further improved the hole-transport properties of the polythiophene-based material. The weak forces present within the molecules that promoted structural coplanarity were analyzed using theoretical simulations. Furthermore, the grazing incidence wide-angle X-ray scanning (GIWAXS) results indicated that PDPP-5Th features high crystallinity, which is favorable for efficient carrier migration within and between polymer chains. The material showed hole transport properties as high as 0.44 cm^2^ V^−1^ s^−1^ in conductivity testing. Our investigations demonstrate the great potential of this polymer material in the field of optoelectronics.

## 1. Introduction

Progress in the development of organic semiconductors as the core of performance modulation of organic field-effect transistors (OFETs) has been a great achievement in the last decade, which mainly consists of the study of the functional π-conjugated small molecules and polymers [1,2,3]. Polymer semiconductor materials are distinguished from small-molecule compounds not only by their molecular weight, which tends to result in a significant lowering of the energy gap between the frontier orbitals, but more importantly by the ease with which they can be processed with a solution method [4]. The process of preparing devices from small molecules usually requires vapor deposition, which frequently involves heating and consumes significant energy. Polymers offer efficient film formation and good chemical modification, making them potential device candidates for large-area processing to prepare organic transistors in high volume [5,6]. Three types of design strategies have been developed and extensively studied for the flexibility to modulate material energy levels and to significantly increase the diversity of molecular structure libraries, which can be briefly classified into all-acceptor, all-donor, and donor–acceptor types [7]. All-acceptor polymer arrangements are systems that contain only structural units with strong electron-withdrawing capabilities and are typically designed and explored for n-type materials [8,9]. On the other hand, the all-donor type can be used for hole-transporting p-type materials due to the electron-donating ability of components in their structural composition. The development of P-type materials is necessary as an integral part of logic circuits [10]. Oligomers and polymers of conjugated thiophene have attracted attention as excellent donor units due to their facile synthesis approach and strong electron-donating ability [11,12]. Pioneering research on the synthesis of polythiophene and its application in transistors dates back to 1980 and 1986, respectively, and it is currently used in a wide range of applications, including organic photovoltaics, organic light-emitting diodes, sensors, electrochromic displays, and many other fields [13,14]. The solubility of polythiophene decreases significantly after its molecular weight reaches a certain level, which limits its processing properties and has thus become a hot research topic [15,16]. The appropriate insertion of solubilizing groups (e.g., alkyl chains, alkoxy chains, etc.) is an effective approach to dramatically improve the solubility of materials. Typical structures have been developed, such as the star molecule poly(3-hexylthiophene), (P3HT) [17]. Benedikt et al. reported that P3HT-based systems showed a moderate mobility of 10^−2^ cm^2^ V^−1^ s^−1^ [18]. Nevertheless, this molecule has its own drawback, which is related to the regioisomers caused by the different constructions of the head–tail, head–head, and tail–tail linkages. One way to further improve the performance of such materials in devices is to minimize the number of regional isomers in the material system [13,19,20]. Therefore, the direct attachment of polythiophene to solubilizing groups through chemical structure modification strategies is logical, as it neither affects the effective conjugation length of the material nor leads to an increase in the number of isomers.

**Figure 1:**
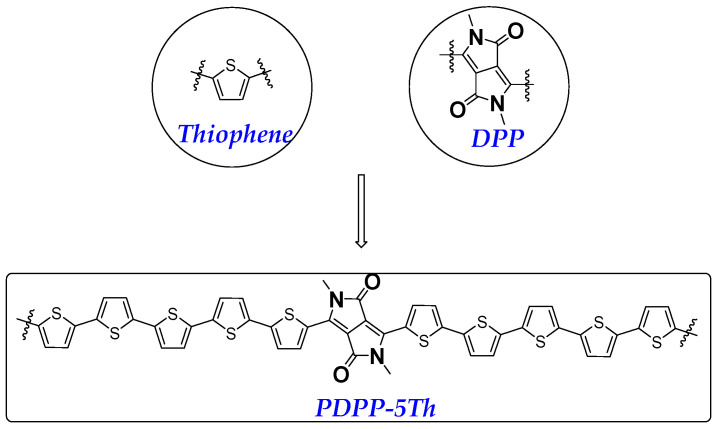
Molecular structure design strategies for target compound with polythiophene units.

The objective of this research was to introduce diketopyrrolopyrrole (DPP) units with long alkyl side chains into a polythiophene molecular system to prepare solution-processable transistor materials with high carrier mobility [21]. Zhang et al. reported polymeric materials based on DPP and thiophene in 2018, which showed an average hole mobility of 0.22 cm^2^ V^−1^ s^−1^ [22]. Geng et al. further reported the control of charge mobility by modulating the molecular weight of the polymer after replacing part of the thiophene with the strong electron donor selenophene [23]. Simultaneously, the DPP itself is an electron acceptor unit, which can further significantly improve the carrier mobility and device performance of the material through donor–acceptor interactions within the main chain [24]. Kim et al. reported polymers based on the composition of DPP and thieno[3,2-b]thiophene, which enabled direct line printing transistors [25]. Similarly, Tsuyoshi et al. also optimized the performance of devices by adjusting the molecular weight of a DPP and thiophene based polymer [26]. Recently, large-area oriented films based on DPP-type polymers prepared via the ribbon-shaped floating film transfer (FTM) technique have also been reported, fully demonstrating the importance of DPP as a moiety [27]. The molecular structure of the donor–acceptor type is illustrated in Figure 1. The macromolecule PDPP-5Th can be prepared via the Stille coupling method, which consists of a tiny fraction of DPP and polythiophene units.

## 2. Results

### 2.1. Synthesis and Characterization of Material PDPP-5Th

**Figure 2:**
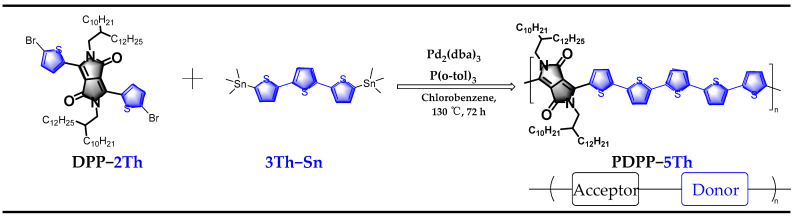
Synthesis condition and route for the π-conjugated polymer of PDPP-5Th.

The preparation of polymer PDPP-5Th is shown in Figure 2, which relies on the monomer 3,6-bis(5-bromothiophen-2-yl)-2,5-bis(2-decyltetradecyl)-2,5-dihydropyrrolo[3,4-c]pyrrole-1,4-dione (DPP-2Th) with monomer 5,5″-bis(trimethylstannyl)-2,2′:5′,2″-terthiophene (3Th-Sn) under palladium catalysis. The specific synthesis procedure is outlined in Section 3. The monomer DPP-2Th contains a DPP unit that is directly attached to the α-position of the thiophene (Th) ring via a single bond. The carbonyl functional group makes it an excellent electron acceptor unit, while its alternating single- and double-bond structure ensures its effective conjugation length. An extremely long aliphatic chain with 24 carbons was chosen for the side chain part, which fully facilitates its solubility in organic solvents. On the other hand, the triple thiophene monomer containing bis-trimethyltin can easily undergo a Stille coupling reaction with monomer DPP-2Th. Crude products containing impurities such as oligomers or catalyst ligands can be purified via Soxhlet extraction [28,29]. The target product shows excellent solubility in chlorinated solvents such as dichloromethane, chloroform, and chlorobenzene. The DPP monomer is a brilliant red pigment dye, and the polymer appears as a deep blackish green powder. Figure S1 in the Supplementary Materials shows the infrared (IR) spectrum of the polymer with its typical carbonyl and alkyl chain peaks appearing near 1660 cm^−1^ and 2848–2917 cm^−1^, respectively. Based on high-temperature gel permeation chromatography, the number average molecular weight (Mn) and the weight average molecular weight (Mw) of the polymer were found to be 33.4 k and 127.9 k, respectively, which correspond to a polydispersity index (PDI) of 3.8. The average minimum repeating units contained in the polymer chains are 27 and 104 based on Mn and Mw speculation. The results of the elemental analyses are shown in Table 1. The experimental values for the three elements are close to the theoretical values, with an overall variation of around 2% [30]. Figure S2 illustrates the thermal decomposition curve of the material. The material presented good stability up to 380 °C, with a mass loss of 5% at approximately 390 °C. The high thermal stability facilitates the subsequent optimization of the performance of devices based on this polymer film by adjusting the annealing temperature.

### 2.2. Density Functional Theory (DFT) Calculation

**Figure 3:**
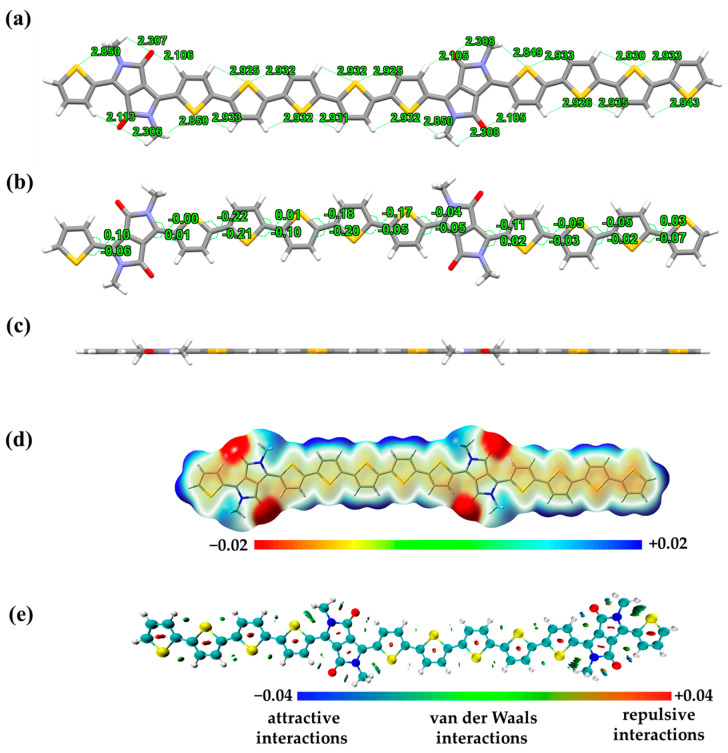
(**a**,**b**) The intramolecular distance and the dihedral angles; (**c**) side view of the dimer; (**d**) electrostatic potential surface of the dimer; (**e**) intramolecular noncovalent bonding interactions analysis of the methyl-substituted dimer.

In order to further characterize the intramolecular architecture of the polymer, DFT calculations derived from B3LYP-D3/6-311g [31,32] on the basis of Gaussian 16 [33,34] were used. The purpose of using short methyl chain substituted dimers as samples for the calculations was to reduce the computational cost and time [35,36,37]. Some of the intramolecular distances and dihedral angles are shown in Figure 3a,b, respectively. The DPP unit consists of two five-membered fused rings and exhibits good coplanarity, with the oxygen atom of the carbonyl group separated from the hydrogen atom on the neighboring thiophene by a distance of 2.1 Å. The distance between the S atom of thiophene and the H atom on the neighboring thiophene is on average 2.9 Å. The dihedral angle between the thiophene groups is extremely small, which results in the material as a whole exhibiting an excellent planarity (Figure 3c). The dihedral angle between the adjacent thiophenes is small, within 0.2°. The torsion angle between the thiophene and the DPP unit in the optimal configuration is close to 0.1°, which shows the coplanarity within the material. Well-defined stacking is crucial for the migration of the carriers. The electrostatic potential diagram of the molecule in Figure 3d reveals that its carbonyl fraction group is strongly electron-withdrawing, whereas the periphery of the polythiophene system exhibits electron-donor feature. It also indicates that the potential of this material for transporting holes is superior to that of electrons. The analysis of intramolecular noncovalent bonding interactions was used to investigate the reasons for the conformational stability, and the results are shown in Figure 3e [38]. There is a pronounced attraction between the oxygen atom of the carbonyl group and the hydrogen atom of the neighboring thiophene. At the same time, there is a weak S...H force between the thiophene and the methyl hydrogen atom. These two interactions ensure that the thiophene unit can form an intramolecular conformational lock without random rotation even when it is linked to the DPP by a single bond. The maintenance planar interaction between thiophene and thiophene relies mainly on the mutual attraction of the S atoms with the adjacent H atoms, which are van der Waals attractions. The spatial atomic coordinates of the dimer are shown in Table S1.

**Figure 4:**
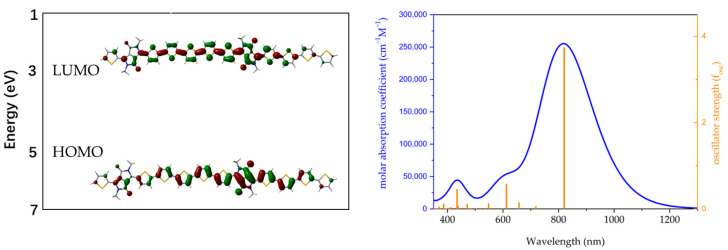
(**left**) HOMO and LUMO map; (**right**) simulated UV–Vis spectra of methyl-substituted dimer of PDPP-5Th.

The frontier orbital maps of the π-conjugated dimer are shown in the left panel in Figure 4. Both HOMOs and LUMOs are completely delocalized on the DPP acceptor group and the π-conjugated thiophene donor segment of the main chain. The HOMOs primarily occupy the double bonds of the molecule along the long-axis direction, whereas the LUMOs are predominantly distributed on the single bonds. The HOMO and LUMO energy levels turn are 5.06 eV and 3.36 eV, respectively, which correspond to an energy gap of 2.00 eV. The simulation orbital maps of HOMO−1 (LUMO−1) and HOMO−2 (LUMO−2) are shown in Figure S3. The simulated UV–Vis absorption in the right panel in Figure 4 shows that the material exhibits a double absorption band with a maximum absorption peak at 819 nm. The material features a strong molar absorption coefficient and a large oscillator strength (f_osc_) of 254.8 k and 3.74, respectively, which corresponds to the linear-conjugate architecture of the material [39].

### 2.3. Photophysical and Electrochemical Properties of PDPP-5Th

**Figure 5:**
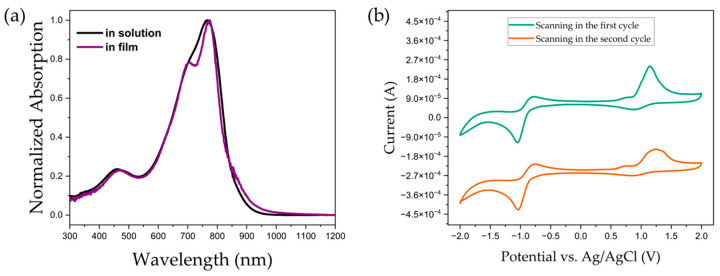
(**a**) Representative absorption of PDPP-5Th in solution and film states. (**b**) Two sequential redox characteristic of PDPP-5Th (0.1 V/s).

To investigate the optical absorption properties of the polymer, absorption measurements of ultraviolet–visible (UV–Vis) in its solution and thin film states were carried out. The material showed absorption in the range of 300–1000 nm, which is similar to the absorption characteristics of most materials based on donor–acceptor architectures. Figure 5a reveals the photophysical properties of the material, which presents a dual absorption band in both states. The absorption bands located at 300–500 nm are relatively weak, with peaks at 459 nm and 465 nm, respectively. The high-energy absorption at short wavelengths is caused by π–π transmission. The absorption at 600–900 nm is stronger and is caused by intramolecular charge transfer. Unlike the solution state where there is only one peak at 765 nm, the film state exhibits a peak at 775 nm, accompanied by a pronounced shoulder. The ratio of the shoulder peak to the maximum absorption peak corresponds to the 0–0 and 0–1 peaks, respectively, and tends to represent the degree of stacking of the material in the thin-film state. The positions of the onset absorption (λ_onset_) peaks appear at 905 nm and 925 nm in the solution and film phases, respectively, and this redshift is also associated with stronger intermolecular π–π stacking in the film. The energy band gap of the materials calculated based on the ratio of 1240 to λ_onset_ is 1.37 eV and 1.34 eV, respectively. The actual test results are closer to the theoretically calculated absorptions in terms of the shape of the curve and the location of the maximum absorption peak, which to some extent also reflects the accuracy of the simulation process.

In order to calculate the energy levels of the material and its oxidation and reduction behavior, we performed cyclic voltammetry measurements on its thin films, as shown in Figure 5b. The green part in the figure below shows the redox curve of the first forward scan, and the overall shape and peak position are not much different from the results of the second cycle scan. This indicates that the material features redox stability. The polymer shows distinct oxidation and reduction peaks at 1.15 V and −1.05 V, respectively. According to the results of the first cycle scan, the positions of the onset absorption and reduction peaks are 0.89 V and −0.84 V, which correspond to the HOMO and LUMO energy levels of the material of 5.29 eV and 3.66 eV, respectively. The oxidation curves are quasi-reversible, while the reduction characteristics demonstrate reversible properties. The energy bandgap of the material calculated from the difference between the HOMO and LUMO energy levels is 1.63 eV, which is dissimilar to that obtained by the photophysical method. The energy levels and bandgap obtained from electrochemical tests were different from those calculated from theoretical simulations, with the main inaccuracy coming from the different conjugation lengths of the samples.

### 2.4. Conductive Properties of OFET Device

**Figure 6:**
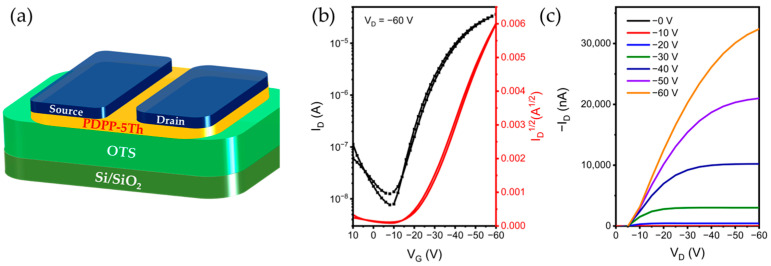
(**a**) The BG/TC OFET architectures; (**b**) transport and (**c**) output characteristics of OFET at annealing temperature of 180 °C.

We fabricated PDPP-5Th-based OFET devices with a bottom gate top contact (BG/TC) structure to measure the charge transport and conductive behavior of the material, and the corresponding device structure is shown in Figure 6a. The identical rotational speeds were aimed at taking a single variable to reduce the complexity of optimizing the performance of the material. The thickness of the polymer film at a speed of 2000 rpm was approximately 40 nm, which was characterized by atomic force microscopy (AFM) testing and is shown in Figure S4. Figure 6b,c display the characteristic transfer and output plots of the best-performing transistors at an annealing temperature of 180 °C. The polymer showed excellent p-type transport properties with a maximum hole mobility of 0.44 cm^2^ V^−1^ s^−1^, which is higher than that of the corresponding polythiophene-type material. Polymers do not show significant electron migration properties and are therefore unipolar conductive transport materials. The transfer curve (red line) exhibited good overlap by testing the process from 10 V to −60 V and back to 10 V, which indicated that the material suffered from small charge traps and low hysteresis. The largest difference between the on and off states (I_ON_/I_OFF_) is 10^4^, which indicates the device’s favorable switching properties. Different annealing conditions affect the electrical conductivity of the material, so we tried four conditions, and their corresponding results are shown in Figure S5. The maximum and average mobility of the polymer at an annealing temperature of 160 °C were the lowest among the four conditions (Table 2).

### 2.5. Thin-Film Surface Morphology

**Figure 7:**
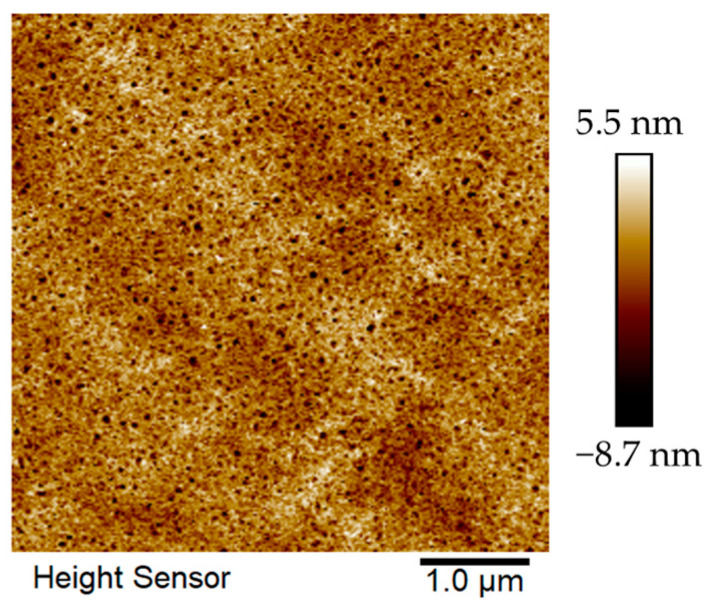
The height images of PDPP-5Th film from chlorobenzene solution annealed at 180 °C.

It is well known that the microscopic morphology of a film surface plays an important role in the charge transport properties of semiconductors. We thus characterized the microstructure of the films via AFM scanning under the same conditions as those used to fabricate OFET devices (Figure 7). The annealed film showed a fibrous intercalation network and distinct crystalline regions with a root mean square of 2.32 nm. The fibrous reticulated homogeneous structure of the annealed films may lead to good crystallinity, thus improving the device performance of the material. The three-dimensional topography of the film is shown in Figure S6.

### 2.6. Molecular Stacking Characterization

**Figure 8:**
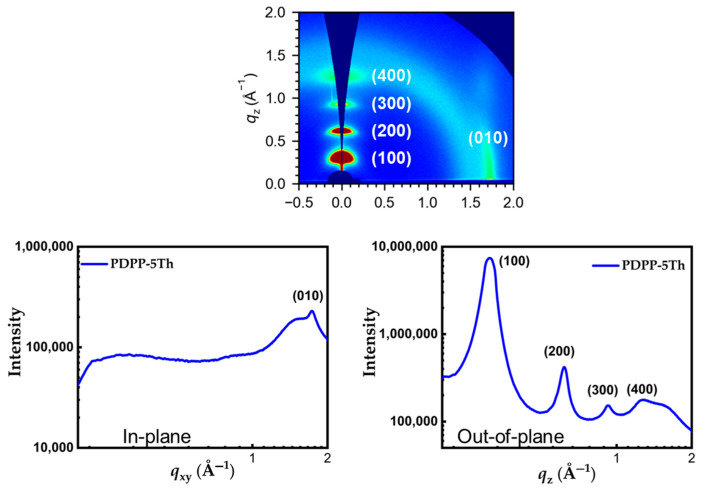
(**top**) Annealed film of 2D-GIWAXS and (**bottom**) 1D-GIWAXS of PDPP-5Th.

The 2D-GIWAXS and 1D-GIWAXS diffraction patterns of the annealed films are presented in Figure 8. The film showed arc-shaped four-level Bragg peaks (h00) in the out-of-plane direction (q_z_ axis), which indicated a high level of crystallinity. In addition, the (010) Bragg peak of the film was located only in the in-plane direction (q_xy_ axis) rather than in the out-of-plane direction (q_z_ axis), which demonstrated that the PDPP-5Th adopts an edge-on packing mode in the film state [40,41]. The edge-on orientation favors efficient charge transport, which explains the excellent hole mobility performance of the material [42]. The π–π stacking distance of PDPP-5Th, estimated from the (010) signal value in the q_xy_ direction, is 3.63 Å, which is similar to the stacking distances of most polymers based on poly(3-hexylthiophene) or polythiophene. Thus, the introduction of DPP motifs with very long alkyl side chains has no significant side effect on the stackedness of the material. High crystallinity promotes tight π–π stacking and thus enhances charge carrier hopping between molecular chains, which effectively increases the hole mobility [43]. The d–d stacking distance of PDPP-5Th was estimated to be 20.26 Å based on the (100) signal value in the q_z_ direction (Table 3) [44].

## 3. Materials and Methods

The materials were as follows: 3Th-Sn and common organic solvents were purchased from SunaTech (Suzhou, China). The precursor DPP-2Th used for the synthesis was prepared using similar synthetic conditions as previously reported [45]. Potassium carbonate (4.15 g, 30.00 mmol, 3.00 eq) was added to a solution of 3,6-di(thiophen-2-yl)-2,5-dihydropyrrolo[3,4-c]pyrrole-1,4-dione (3.00 g, 10.00 mmol) in anhydrous dimethylformamide (DMF, 70 mL) under argon protection. The temperature was raised to 60 °C and maintained for a half hour, after which 11-(bromomethyl)tricosane (9.19 g, 22.00 mmol, 2.20 eq) was added to a three-necked flask. Argon was withdrawn, and the mixture was stirred at 100 °C for 24 h. The DMF solvent was removed after the reaction returned to room temperature, and the organic layer was subsequently extracted with dichloromethane. After removal of the solvent under reduced pressure, the residue was purified via silica gel chromatography with petroleum ether and dichloromethane (3:1) as eluents. 2,5-bis(2-decyltetradecyl)-3,6-di(thiophen-2-yl)-2,5-dihydropyrrolo[3,4-c]pyrrole-1,4-dione (5.88 g, 60.5%) was obtained as an orange solid. It was next mixed with N-bromosuccinimide (2.26 g, 12.69 mmol, 2.10 eq) in 30 mL of chloroform. The reaction was carried out at 60 °C for a half hour and then terminated. After removal of the solvent under reduced pressure, the residue was extracted with dichloromethane and then purified via silica gel chromatography with petroleum ether and dichloromethane (4:1) as eluents. DPP-2Th (6.27 g, 92.0%) was obtained as a claret solid.

For the synthesis of π-conjugated polymer PDPP-5Th, DPP-2Th (200.00 mg, 0.17 mmol, 1.0 equiv), 3Th-Sn (102.50 mg, 0.17 mmol, 1.0 equiv), and tris(o-tolyl)phosphine (P(o-tol)_3_, 3.62 mg, 14.14 µmol) were weighed in a 50 mL Schlenk tube. Afterwards, dry chlorobenzene (10 mL) was added to the system. Argon was passed into the tube for 25 min to completely remove oxygen out of the system. Subsequently, [Pd_2_(dba)_3_] (3.23 mg, 3.53 µmol, 2%) was added. The polymerization process was continuously carried out at 130 °C under stirring for 3 days and the color of the mixture changed to dark green. The purification process of the crude was carried out via Soxhlet extraction. The oligomers were sequentially extracted with methanol, acetone, hexane, and ethyl acetate under reflux conditions for 10 h each. The target product was extracted using chloroform. The fractions were then added dropwise to methanol (180 mL) and pump-filtered to afford a dark green solid.

For characterization, the molecular weight of PDPP-5Th was evaluated via gel permeation chromatography at 150 °C (Agilent PL-GPC220, Santa Clara, CA, USA). Polystyrene-divinylbenzene and polystyrene were applied as the stationary phase and reference, respectively. Trichlorobenzene served as the eluent to ensure sufficient solubility of the long-conjugated chains. A solution with a concentration of 0.1 mg/mL was pumped through the column (2* PLgel 10 μm MIXED-B, Agilent, Santa Clara, CA, USA) at a flow rate of 1.0 mL/min. A thermal stability test with PDPP-5Th powder was conducted under nitrogen (TGA 550, Tainstruments, New Castle, DE, USA). An IR test was conducted by mixing the polymer with potassium bromide in the powdered state (Vertex 70, Bruker, Karlsruhe, Germany). Elemental analysis of PDPP-5Th powder was performed in CHN mode with an organic elemental analyzer (Elementar Vario, Frankfurt, Germany). The photophysical characterization was carried out with a UV–visible spectrometer (Cary 5000, Agilent, USA) on samples in both solution and thin-film states, respectively. In the solution state, the polymer solution ha a concentration of approximately 0.5 mg/mL in chloroform and appeared to be a light green color. The films were prepared via spin coating the aforementioned solutions onto precleaned quartz plates with annealing (1.0 cm × 2.0 cm, 100 °C). Electrochemical testing of the films of the polymer was conducted in an acetonitrile solution that contained a small portion of tetrabutylammonium hexafluorophosphate. The three-electrode system consists of an Ag/AgCl electrode, a glassy carbon electrode, and a platinum electrode, which were utilized as a reference electrode, a working electrode, and a counter electrode, respectively. A 1.0 mg/mL chloroform solution of the polymer PDPP-5Th was pre-prepared, and 5.0 µL of the solution was pipetted onto the working electrode (4 mm of diameter). Subsequently, the solvent was evaporated using the pressure of a wash ball to form a dense film. The LUMO and HOMO energy levels were estimated by extracting data from the potentials at the onset of the reduction peak and oxidation peak, respectively, according to the following equations: E_LUMO_ = −4.80 − (E_red_^onset^ − 0.40) eV and E_HOMO_ = −4.80 − (E_ox_^onset^ − 0.40) eV, where E^onset^ is the value of the oxidation or reduction potential measured with respect to the Fc/Fc^+^ redox pair. AFM (Nanoscope V, Bruker, Germany) was used to measure the morphology of the thermally annealed films prepared under the same conditions as those used for OFET testing.

For OFET preparation, highly doped n-type silicon (Si) wafers and 300 nm of silicon dioxide (SiO_2_) were employed as the substrate. The substrate was further modified via chemical bonding using octadecyltrichlorosilane (OTS), followed by ultrasonic cleaning in deionized water, acetone, and isopropanol for 10 min, respectively. To prepare semiconductor films, PDPP-5Th was predissolved in chlorobenzene (7 mg/mL) and heated overnight at 80 °C with stirring. The corresponding solution was spin-coated onto the substrates (2000 rpm, 60 s) and then annealed in a glove box under nitrogen at 180 °C for a half hour. Gold was deposited as source and drain electrodes (30 nm) via vacuum vapor deposition to complete the device construction.

## 4. Conclusions

In this work, the DPP molecule was inserted into every fifth spaced thiophene unit using a rational design to prepare a π-conjugated polymer with high molecular weight and thermal stability. DPP plays a dual role in this system not only by improving the solubility of polythiophene and the processability of the material but also enhancing the intramolecular donor–acceptor interaction. Theoretical simulations show that the introduction of DPP has no effect on the structural regularity and coplanarity of the material, which facilitates efficient carrier transport. OFET devices fabricated with PDPP-5Th as the core component had an average hole mobility of 0.40 cm^2^ V^−1^ s^−1^, which we attribute to the high crystallinity of the material. Various annealing temperatures have an effect on the carrier mobility of the material, and more thermal tests are needed to enrich this conclusion and continue to improve device performance. Research on this polymer-based material for stretchable and self-healing applications is still ongoing in our laboratory.

## Figures and Tables

**Table 1:** Test results for molecular weight and elemental content of PDPP-5Th.

	Mn	Mw	PDI ^1^	C ^2^	H ^2^	N ^2^
	(Da)	(Da)		(%)	(%)	(%)
PDPP-5Th	33,403	127,947	3.83	71.53	8.68	2.02
Repeating unit	N/A	N/A	N/A	72.85	9.09	2.30

^1^ PDI = Mw/Mn; ^2^ mean value of two tests.

**Table 2:** Carrier performance of PDPP-5Th**-**based OFET device.

Coating Speed (rpm)	Annealing Temperature (°C)	Max Hole Mobilities (cm^2^ V^−1^ s^−1^)	Average Hole Mobilities ^1^ (cm^2^ V^−1^ s^−1^)	Threshold Voltage (V)	I_ON_/I_OFF_
2000	160	0.21	0.16 ± 0.04	−23	10^3^–10^4^
2000	180	0.44	0.40 ± 0.03	−19	10^3^–10^4^
2000	200	0.39	0.34 ± 0.02	−26	10^3^–10^4^
2000	220	0.29	0.22 ± 0.05	−29	10^3^–10^4^

^1^ Average value derived from eight devices.

**Table 3:** The crystallographic information of PDPP-5Th thin film.

Material	In-Plane (010) Peak (Å^−1^)	π-Spacing (Å) ^1^	Out-of-Plane (100) Peak (Å^−1^)	d-Spacing (Å) ^1^
PDPP-5Th	1.73	3.63	0.31	20.26

^1^ Calculated from the Bragg’s equation: 2dsinθ = nλ.

## Data Availability

Data are contained within the Supplementary Material and the article.

## Supplementary Materials

The following supporting information can be downloaded at: https://www.mdpi.com/article/10.3390/molecules29020457/s1, Figure S1: Infrared spectra of polymers PDPP-5Th; Figure S2: The thermal decomposition curve of PDPP-5Th; Figure S3: HOMO−1, HOMO−2, LUMO+1 and LUMO+2 map of the dimer of PDPP-5Th; Figure S4: Thickness plot of the polymer film based on AFM measurements; Figure S5: (top) Transfer and (bottom) output curves of conducting polymers at different annealing temperatures; Figure S6: Three-dimensional view of polymer film under AFM testing; Table S1: Spatial atomic coordinates of the dimer.
