# Supplementary material for: Organic Transistors Based on Highly Crystalline Donor–Acceptor π-Conjugated Polymer of Pentathiophene and Diketopyrrolopyrrole
Source: Molecules. 2024 Jan 17;29(2):457. doi: 10.3390/molecules29020457 (PMC10819643; doi:10.3390/molecules29020457)
Supplement: Supplementary file 1 [file molecules-29-00457-s001.zip › molecules-2820778-supplementary.pdf]

Supplementary Material

Figure S1. Infrared spectra of polymers PDPP-5Th.

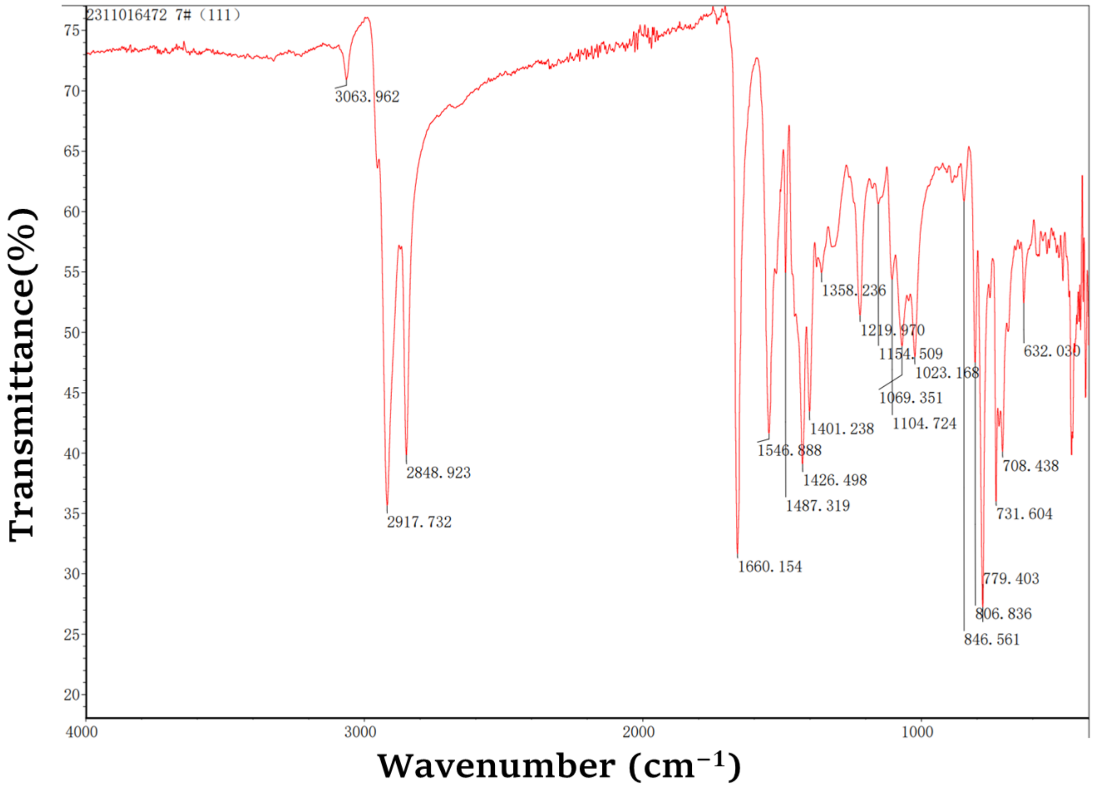

Figure S2. The thermal decomposition curve of PDPP-5Th.

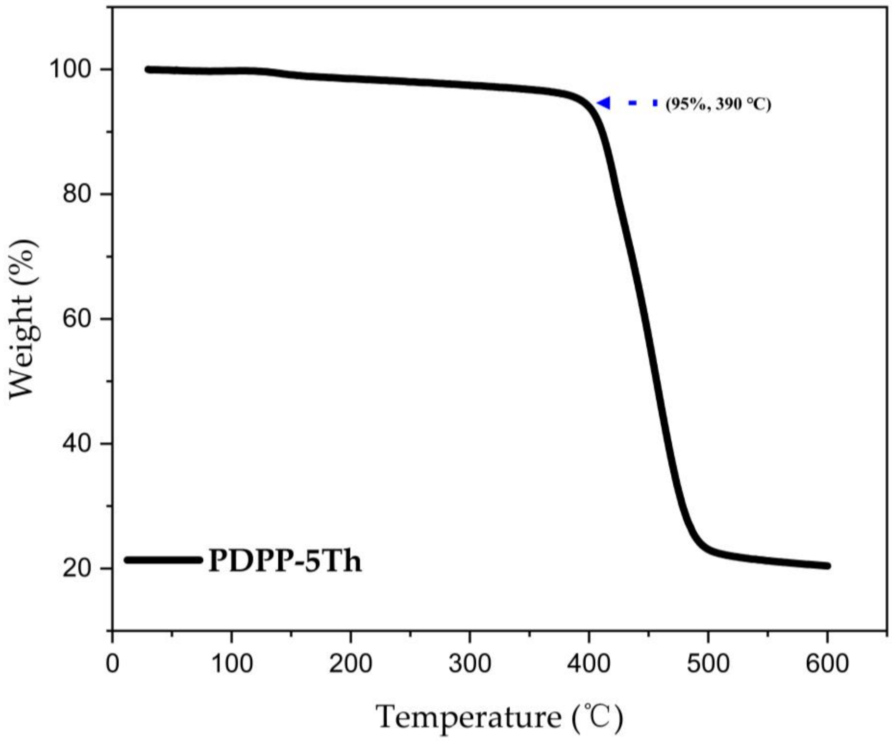

Figure S3. HOMO−1, HOMO−2, LUMO+1 and LUMO+2 map of the dimer of PDPP-5Th.

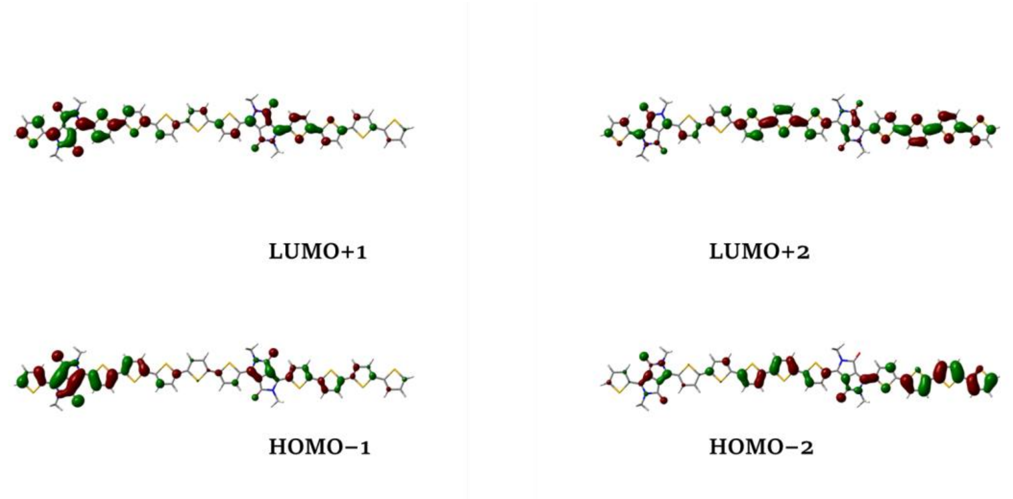

Figure S4. Thickness plot of the polymer film based on AFM measurements

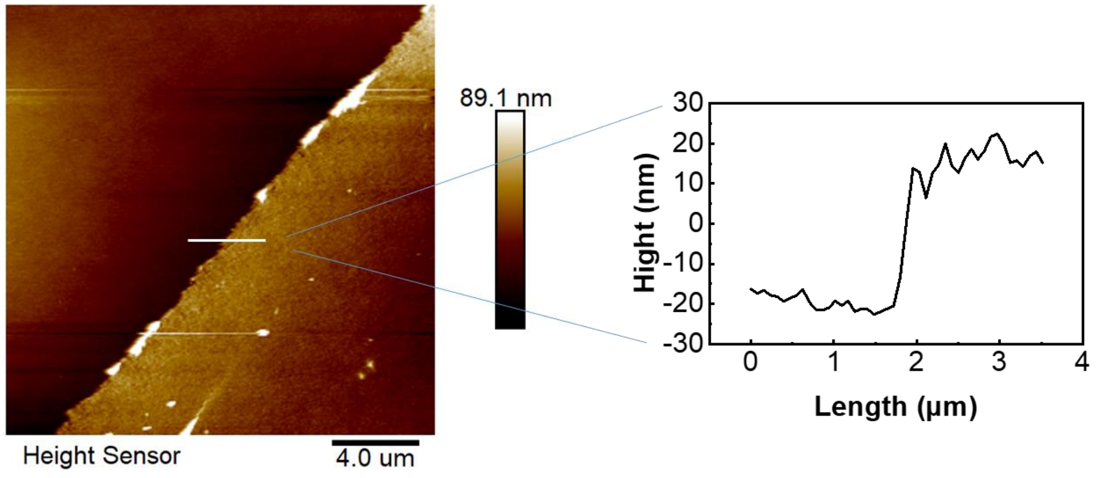

Figure S5. (top) Transfer and (bottom) output curves of conducting polymers at different annealing temperatures.

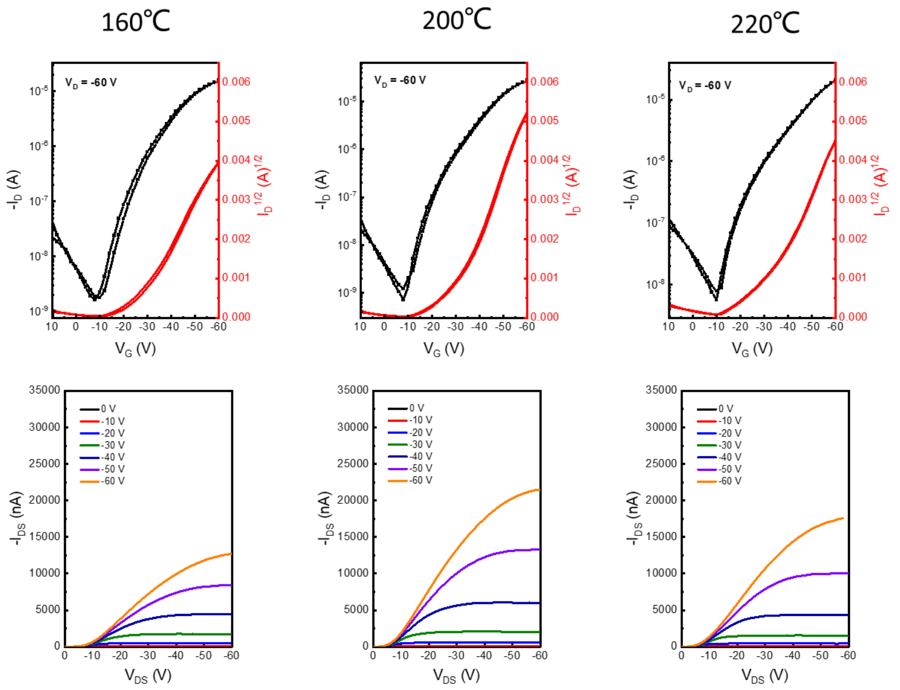

Figure S6. Three-dimensional view of polymer film under AFM testing.

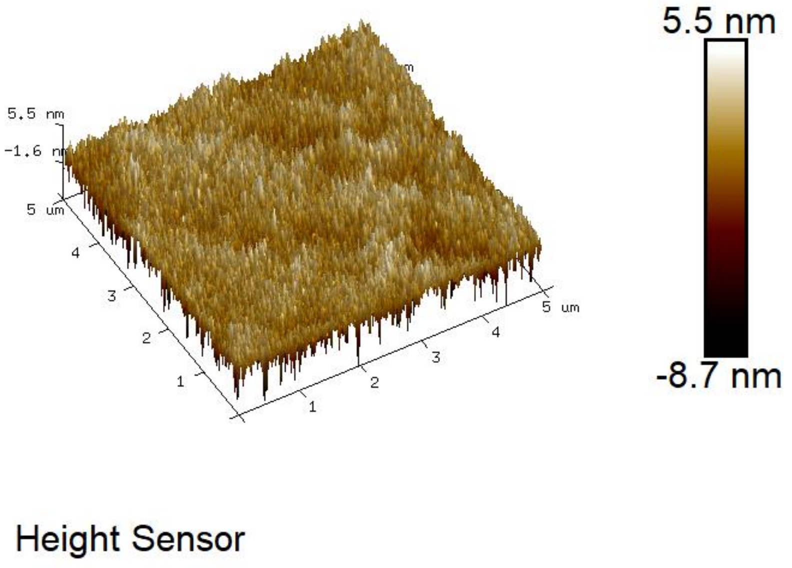

Table S1. Spatial atomic coordinates of the dimer

C	-19.15246900	0.93658000	-0.00535800
C	-18.14683600	-0.03346300	-0.00988800
C	-16.87193500	0.60537600	-0.00201600
C	-17.07471900	2.02680200	0.00786500
N	-18.50754400	2.18189300	0.00542500
C	-17.94416300	-1.45610500	-0.01952900
N	-16.51266300	-1.61182900	-0.01698600
C	-15.86512100	-0.36555300	-0.00643300
O	-16.28957400	3.00501700	0.01697600
O	-18.72936000	-2.43354000	-0.02847700
C	-14.45964300	-0.13678100	-0.00142800
C	-20.56414900	0.70781200	-0.01069300
C	-19.09078700	3.51779200	0.01346700
C	-15.92893600	-2.94723500	-0.02517900
S	-21.83367700	2.02939500	-0.00518100
C	-23.11550900	0.77553100	-0.01756300
C	-22.59670400	-0.48639500	-0.02482300
C	-21.17461100	-0.53208500	-0.02107900
S	-13.18949500	-1.45660500	-0.00815400
C	-11.88140500	-0.19174000	0.00395400
C	-12.43863800	1.07238800	0.01175200

C	-13.84728200	1.10717300	0.00885700
C	-10.51229300	-0.58265900	0.00402000
C	-9.95007300	-1.84141000	-0.00022700
C	-8.53526500	-1.86448400	0.00102900
C	-7.93335800	-0.62411100	0.00631600
S	-9.20104700	0.68960800	0.01010900
C	-6.55103000	-0.27944700	0.00866700
S	-5.28236100	-1.59420200	0.00615600
C	-3.97129000	-0.32172000	0.01074700
C	-4.53297600	0.93657000	0.01368000
C	-5.94848300	0.95970000	0.01253300
C	-2.60109300	-0.71146500	0.01067700
C	-2.04014400	-1.97096100	0.01079400
C	-0.62550600	-1.99451800	0.01044800
C	-0.02200400	-0.75476300	0.01005700
S	-1.29085100	0.55998100	0.01009300
C	1.35895200	-0.40952300	0.00946900
S	2.62446400	-1.71740200	0.01223900
C	3.93804900	-0.44052300	0.00926400
C	3.36695000	0.82356600	0.00669300
C	1.95833000	0.83548100	0.00683500
C	5.33411600	-0.71660300	0.00938900

N	5.94019100	-1.98358800	0.01227700
C	7.37538700	-1.87539300	0.01120300
C	7.62516600	-0.46056400	0.00740700
C	6.37456700	0.22019000	0.00647900
C	8.66587200	0.47664900	0.00393400
N	8.05961800	1.74360300	0.00096000
C	6.62474900	1.63536600	0.00241900
C	8.68736800	3.05900800	-0.00289000
C	5.31258500	-3.29897500	0.01640600
O	5.87091900	2.63796400	0.00036300
O	8.12902000	-2.87834800	0.01347400
C	10.06155800	0.20130800	0.00338400
C	10.63313700	-1.06267300	0.00674500
C	12.04168700	-1.07447900	0.00506400
C	12.64096800	0.17073900	0.00030600
S	11.37549100	1.47841700	-0.00227400
C	14.02191000	0.51641600	-0.00257500
S	15.29170500	-0.79807800	0.00130000
C	16.60122400	0.47361300	-0.00535000
C	16.04025100	1.73256800	-0.00978400
C	14.62503600	1.75573600	-0.00826600
C	17.97275600	0.08399900	-0.00539000

C	18.53594200	-1.17151600	-0.00029600
C	19.95428500	-1.19122500	-0.00213800
C	20.55256800	0.04684300	-0.00872800
S	19.28237900	1.35841100	-0.01314500
C	21.93929400	0.39995700	-0.01224400
S	23.21598100	-0.90848600	-0.00726100
C	24.49495100	0.36078400	-0.01530200
C	23.96203600	1.61348200	-0.02092600
C	22.53288200	1.63861600	-0.01923200
H	-18.25503000	4.21315300	0.02110800
H	-19.69194700	3.69484600	-0.87702600
H	-19.69735800	3.68172400	0.90278600
H	-15.32364700	-3.11193700	-0.91540400
H	-15.32791800	-3.12472900	0.86547300
H	-16.76455700	-3.64293700	-0.03218300
H	-24.14366500	1.08917300	-0.01831000
H	-23.21344600	-1.37170700	-0.03268000
H	-20.59676200	-1.44665700	-0.02591700
H	-11.83261900	1.96558600	0.01973500
H	-14.42904600	2.01955000	0.01416300
H	-10.55110500	-2.73819200	-0.00406300
H	-7.96316900	-2.77989100	-0.00195600

H	-3.93196600	1.83331100	0.01674400
H	-6.51982700	1.87564600	0.01447500
H	-2.64198600	-2.86712300	0.01122900
H	-0.05420700	-2.91051300	0.01039400
H	3.97892900	1.71594400	0.00458100
H	1.38226900	1.74835200	0.00477800
H	9.29511300	3.20744900	-0.89425300
H	7.87547700	3.78225300	-0.00389600
H	9.29684300	3.21181400	0.88655400
H	6.12471900	-4.02198200	0.01817000
H	4.70443800	-3.44725900	0.90756600
H	4.70367400	-3.45250800	-0.87333900
H	10.02130200	-1.95520400	0.01022300
H	12.61809500	-1.98707500	0.00718600
H	16.64209300	2.62864100	-0.01406900
H	14.05373800	2.67181300	-0.01136800
H	17.93741300	-2.07003100	0.00474900
H	20.52763500	-2.10583700	0.00134300
H	25.52539400	0.05637100	-0.01509200
H	24.56790700	2.50660600	-0.02620500
H	21.95962500	2.55339000	-0.02313100
